# Method for retrospective, respiratory-gated, anatomical optical coherence tomography for airway wall elastography

**DOI:** 10.1117/1.JBO.30.12.124502

**Published:** 2025-08-05

**Authors:** Srikamal J. Soundararajan, Yinghan Xu, Nicusor Iftimia, Carlton J. Zdanski, Amy L. Oldenburg

**Affiliations:** a University of North Carolina at Chapel Hill, Biomedical Research Imaging Center, Chapel Hill, North Carolina, United States; b University of North Carolina at Chapel Hill, Department of Biomedical Engineering, Chapel Hill, North Carolina, United States; c Physical Sciences Inc., Andover, Massachusetts, United States; d University of North Carolina at Chapel Hill, Department of Otolaryngology/Head and Neck Surgery and Pediatrics, School of Medicine, Chapel Hill, North Carolina, United States; e University of North Carolina at Chapel Hill, Department of Physics and Astronomy, Chapel Hill, North Carolina, United States

**Keywords:** optical coherence tomography, endoscopic optical coherence tomography, airway compliance, dynamic airway imaging, optical coherence elastography, bronchoscopy, upper airway, biomechanical properties

## Abstract

**Significance:**

Airway wall elastography (AWE) is promising for evaluating upper airway obstructive disorders and airway injuries. Technologies for AWE based on endoscopic optical coherence tomography (OCT) provide micron-scale resolution to capture airway wall deformations during tidal breathing. Combined with an intraluminal pressure probe, these technologies can provide quantitative AWE as part of a routine bronchoscopy exam. However, scan times must be of short duration to mitigate risk.

**Aim:**

Our objective is to reduce the scan time necessary to perform OCT elastography over a 50 mm length of the airway wall to less than 1 min.

**Approach:**

We introduce an innovative, 4D OCT imaging technique that scans in a sawtooth pattern to revisit each axial position of the airway over a diversity of respiratory phases. An anatomical (long-range) OCT system capable of capturing cross-sections of the upper airway was employed in conjunction with an intraluminal pressure catheter. Scanned data are retrospectively sorted into axial bins with high- and low-pressure thresholds used to compute cross-sectional compliance (CC) within each bin across the length of the upper airway.

**Results:**

4D OCT was tested in simulation, on rigid and deformable samples, and on *in vivo* pigs undergoing bronchoscopy. A precise CC measurement with a 0.5 mm sampling resolution over a 50 mm scan length in under 42 s was achieved.

**Conclusions:**

The retrospective, respiratory-gated 4D aOCT scanning method is a minimally invasive technique for measuring airway wall CC. The method exhibited high precision in controlled models, effectively detected elastic heterogeneity, and yielded clinically relevant results in *in vivo* pigs.

## 1 Introduction

Endoscopic optical coherence tomography (OCT) is of increasing interest for upper airway imaging as it is minimally invasive and able to be deployed through most conventional bronchoscopes. OCT also provides histologic-like imaging of subsurface tissue features, which in the upper airway has been beneficial for studying laryngeal pathologies,^1^ including subglottic stenosis.^2^ Long-range OCT systems, i.e., OCT systems with >10  mm working distance (compared with 2 to 3 mm in conventional OCT systems), have been designed for upper airway endoscopy to capture the shape of the airway lumen in addition to its subsurface features.^3^^–^^7^ Although the airway lumen is visible by other imaging modalities such as CT and MRI, OCT affords higher resolution (on the order of 10  μm), which may aid in studies of subglottic stenosis,^2^^,^^4^ sleep apnea,^8^ or other upper airway disorders.

In addition, the high resolution afforded by OCT offers sufficient sensitivity to detect pressure-induced deformation of the airway wall during respiration (pseudo pressure-volume curves), as shown in a comparative study of dynamic OCT and CT of *in vivo* pigs.^9^ Taking this one step further, one can use intraluminal pressure (stress) and dynamic changes in OCT-based luminal cross-sectional area (CSA) (strain) to quantify the cross-sectional compliance (CC) of the airway wall along its length^10^ or OCT-based airway deformation versus pressure to measure local compliance,^11^ both of which constitute a form of optical coherence elastography (OCE). Airway wall mechanics are important in many disease processes, such as inhalation injury, and obstructive airway disorders such as airway stenosis and sleep apnea. In one study, Bu et al.^12^ showed that OCE could detect heat injury-associated changes in airway wall cross-sectional compliance. Although an earlier study showed that OCE could detect elastic properties of the central airways in obstructive lung disease,^13^ for real-time applications, long-range, and swept-source OCT systems are preferred^3^ as they offer the speed required to capture airway deformation during respiration.

Currently, endoscopic OCE of the upper airway is primarily limited by the scan time needed to sample the entire airway dynamically, i.e., to provide (3 + 1)D imaging (three spatial dimensions and one temporal dimension) so that airway wall mechanical properties can be mapped over its entirety. A limiting factor is that each airway position must be sampled during the low- and high-pressure phases of the respiratory cycle. Interestingly, a prior study employed a respiratory-gated method with OCT to minimize motion artifacts in quantifying airway geometry in healthy human volunteers, and the results emphasized the need to separate images by respiratory phase.^14^ In that study, 2 min scans were employed at each of two anatomical locations. In this paper, we propose a novel, 4D, retrospectively gated OCT method to speed up acquisition such that a significant portion of the upper airway (in this case, comprised of 100 anatomical locations) can be sampled in less than a minute.

Other modalities offer methods for airway elastography and respiratory-gated imaging. Magnetic resonance elastography employs MRI and mechanical vibrations for creating elastograms of the lung but has limited availability in clinical practice and requires patients’ cooperation during breath-holding.^15^ Endobronchial ultrasound elastography is performed using a probe introduced into the airway through a bronchoscope; compared with OCT, it has a limited spatial resolution and has primarily been used as a diagnostic tool in treating lung cancer.^16^ Respiratory-gated CT or CINE CT techniques are of increasing popularity but require long-duration exposure to radiation and have limited spatial resolution to capture airway deformation.^9^^,^^17^ At this time, there exist very few investigations on measuring upper airway elastography or on capturing airway deformation during multiple respiratory cycles to quantify airway compliance. Unlike other noninvasive modalities, aOCT is limited to collecting one cross-section at a time. As such, we propose a retrospectively gated method where the catheter scans back and forth to collect images at different phases of the respiratory cycle at each cross-section. This is achieved by scanning with a sloped sawtooth waveform at a frequency different from the respiratory rate to ensure that cross-sectional images are collected at different phases of the respiratory cycle at each cross-section along the airway. Our scanning method requires that, at each position, there exists at least one CSA of the airway lumen each at a pressure corresponding to the inspiration and expiration phases of respiration. Our approach is then to retrospectively bin the data by position and analyze CSA as a function of pressure within each bin to extract CC at each position in the airway.

We hypothesized that the proposed retrospective 4D scanning method is capable of capturing the airway geometry and CC along the length of the upper airway precisely. In this paper, first, we show simulated OCT scans to determine appropriate control parameters for the sawtooth waveform. We then validate the method using a static 3D structured target and then two deformable objects: a uniform cylinder and a structured balloon. Finally, we perform 4D scanning and subsequent elastography (in the form of CC versus position) of the upper airways of *in vivo* pigs. Importantly, this method is capable of capturing airway wall elastography (AWE) over a scan length of 50 mm in under 42 s. This short scan time makes the technology suitable for a wide variety of applications in humans where it can be used as an adjunct to interventional bronchoscopy.

## 2 Methods and Materials

### 2.1 Proposed (3 + 1)D Respiratory-Gated Scan Method

**Fig. 1:**
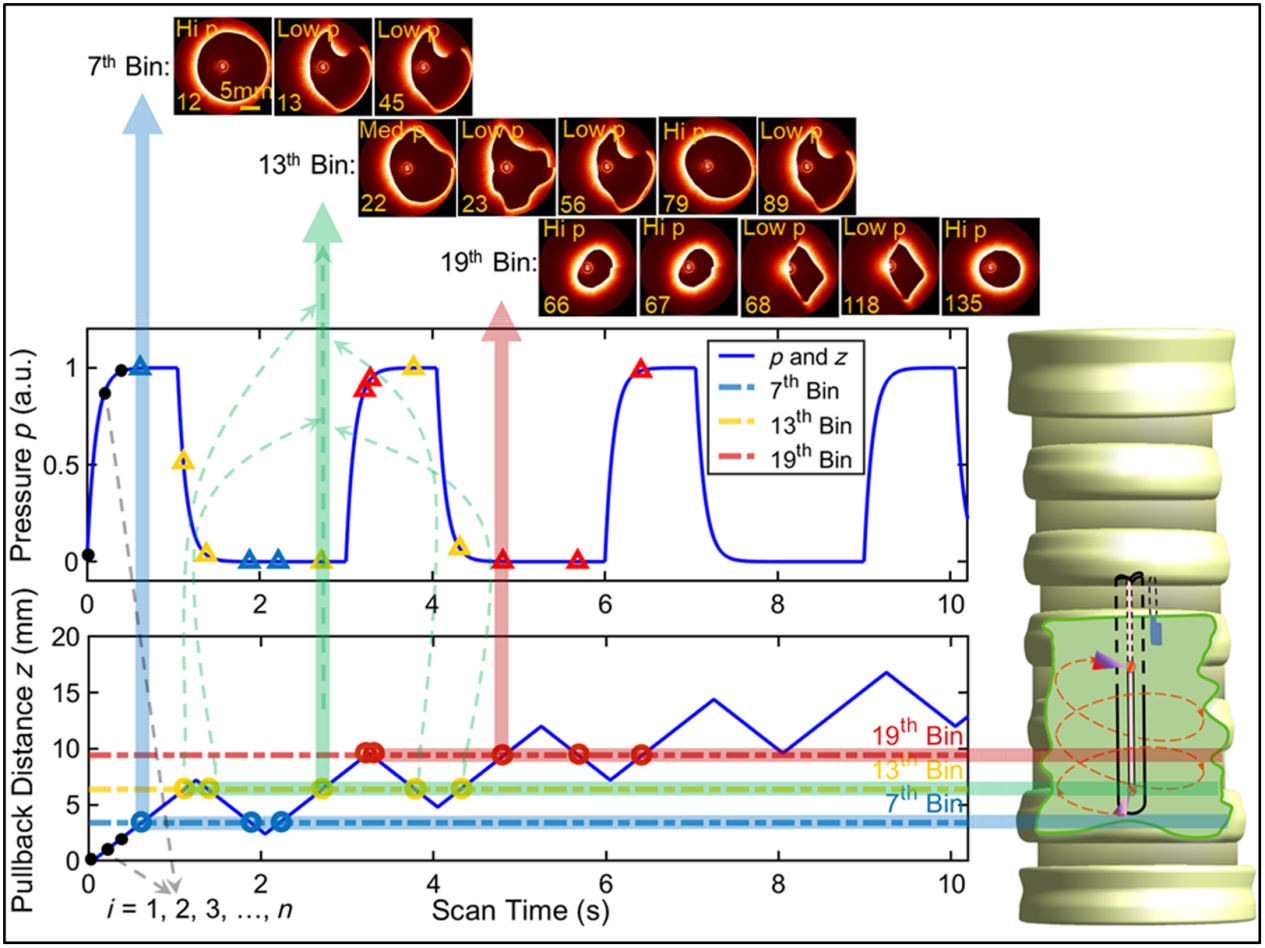
Simulation of retrospectively respiratory-gated aOCT using a sloped sawtooth waveform. The top plot depicts a simplified simulated pressure waveform in time at 20 BPM with I:E ratio of 1:2. The bottom plot depicts an example trajectory of the aOCT imaging catheter pullback distance (z) in time with sawtooth frequency $f_{\text{wfm}}=0.5\;\mathrm{Hz}$, speed v=6  mm/s, and α=0.6. aOCT (and associated pressure) are collected continuously at 20 Hz ($i=1,2,3\ldots ,N_{\text{frm}}$). The binning process is depicted for a bin width Δz=0.5  mm, where aOCT frames are sorted within every Δz=0.5  mm window along the airway. The blue, green, and red highlighted horizontal sections depict selected bins j=7, 13, and 19 defined by bin edges (j−1)*Δz, j*Δz to illustrate the process. aOCT frames and corresponding pressure values lying within each blue, green, and red bin can be seen in the example images from 4D aOCT balloon scan (top) and pressure curves (middle), showing how each bin contains at least one frame, each collected at high and low pressure (with correspondingly high and low CSA); in one case, the pressure is intermediate (labeled “Med p”).

To measure airway wall elastic properties, we must simultaneously monitor airway wall deformation (strain) and luminal pressure (stress) during respiration (which cycles the stress). Here, we quantify the CC, defined as the change in airway lumen cross-sectional area (ΔCSA) per unit change in pressure (Δp), by capturing the lumen shape with aOCT while simultaneously measuring pressure at multiple phases of the respiratory cycle. Because respiratory pressure is somewhat bimodal in time, with high and low pressures during inspiration and expiration, respectively (see, e.g., Fig. 1), CC measurements are more accurate when multiple aOCT frames are collected during both inspiration and expiration. One could measure CC over the length of the airway (z) by collecting aOCT frames over multiple respiratory cycles per z position, then translating the imaging catheter to a new z position and repeating. However, scanning in this way is time-prohibitive, especially for *in vivo* studies.

Here, we propose to speed the process by scanning in z with a pattern that allows for multiple z positions to be captured during each phase of the respiratory cycle while returning to the same z position multiple times over multiple cycles. Retrospectively sorting the aOCT image frames within user-defined bins in the *z*-axis then allows for CC to be estimated from the slope of the CSA versus p registered to each bin. Because the expiratory phase is longer than the inspiratory phase, a limiting factor is collecting sufficient inspiratory frames within each bin while selecting an appropriate bin size (Δz) that balances the need for spatial resolution (small bins) against the need for short scan time (large bins).

To accomplish this, we propose to use a sloped sawtooth trajectory of the imaging catheter in *z* to concurrently capture CSA and p across the entire region under examination. The waveform is defined by several parameters: $f_{\text{wfm}}$ is the sawtooth (peak-to-peak) frequency, $v=v_{\mathrm{back}\;}=v_{\text{forward}}$ is the pullback/push forward speed, $f_{\text{rot}}$ is the frequency of the mechanical scanner rotation (which sets the image acquisition rate and is fixed at 20 Hz in these studies), and α is the fraction of the time in pullback per sawtooth cycle. The latter is set to a value >0.5 and <1 to adjust how much longer the scanner pulls back than pushes forward (and consequently its net advance in z per sawtooth cycle), so that the overall motion of the catheter is to pull back across the length of the airway (over total distance $z_{\text{tot}}$), as shown in the bottom plot of Fig. 1. To ensure that multiple aOCT frames are captured at both inspiratory and expiratory phases of the respiratory cycle, the sawtooth parameters need to be selected depending upon the anticipated respiratory rate in BPM (respirations per minute) and the anticipated inspiratory:expiratory (I:E) ratio. We typically also want to minimize the total scan duration $T_{\text{tot}}$, which can be written in terms of the sawtooth parameters and total pullback distance as

$$T_{\mathrm{tot}}=\frac{z_{\mathrm{tot}}}{v(2\alpha -1)}.$$ (1)

The number of aOCT frames captured, $N_{\text{frm}}$, is then given by

$$N_{\mathrm{tot}}=T_{\mathrm{tot}}f_{\mathrm{rot}}.$$ (2)

The distance $z_{\text{tot}}$ is divided into bins of width Δz, and the collected aOCT image frames and corresponding pressure data (sampled at 5 kHz and time-averaged within each frame) are sorted into these bins retrospectively.

Numerical simulations are employed to determine the sawtooth scanning parameters $f_{\text{wfm}},v$, and α appropriate for a given experiment (given BPM, I:E ratio, and desired $z_{\text{tot}}$, Δz, and $T_{\text{tot}}$). The simulations employ a simplified waveform of the pressure cycling during respiration based on BPM and I:E ratio. The sawtooth parameters are chosen such that the acquired data will contain at least one snapshot of CSA during both inspiration and expiration for any chosen bin within the scan region. Figure 1 shows an example numerical simulation illustrating the binning process for clinically practical values, with BPM=20, $f_{\text{wfm}}=0.5\;\mathrm{Hz}$, v=6  mm/s, and α=0.6, yielding a total scan time of $T_{\text{tot}}=42s$ with $N_{\text{frm}}=840$ for total scan distance $z_{\text{tot}}=50\;\mathrm{mm}$. For clarity, the simulation is only plotted over the first 10 s of the scan in Fig. 1. A bin size of Δz=0.5  mm was chosen, which represents the sampling resolution of the CC measurements. The sawtooth waveform (blue) represents the catheter trajectory during the aOCT scan, and the simulated pressure waveform (orange) is plotted over the same time. Blue, green, and red circles and triangles represent corresponding aOCT and pressure data. The horizontal and vertical dashed lines highlighted with blue, green, and red are presented as a guide for tracing the example bins j=7, 13, and 19 for demonstration purposes, respectively. For example, the green highlighted region, bin 13, contains the aOCT images sampled at the same z location at time indices time i=22, 23, 56, 79, and 89. One of these time points (i=79) is collected at high pressure, three (i=23, 56, and 89) are collected at low pressure, and the first one (i=22) is collected at an intermediate pressure between inspiration and expiration. CSA is determined from the segmentation of the lumen area for each aOCT image, and a linear regression of CSA versus pressure within each bin is used to compute CC according to the slope of the regression line. However, in practice, the data values at intermediate pressures are noisy due to the rapid change in pressure. To mitigate the noise, we omitted from the regression any frames collected within this intermediate range of pressures.

### 2.2 Data Processing

**Fig. 2:**
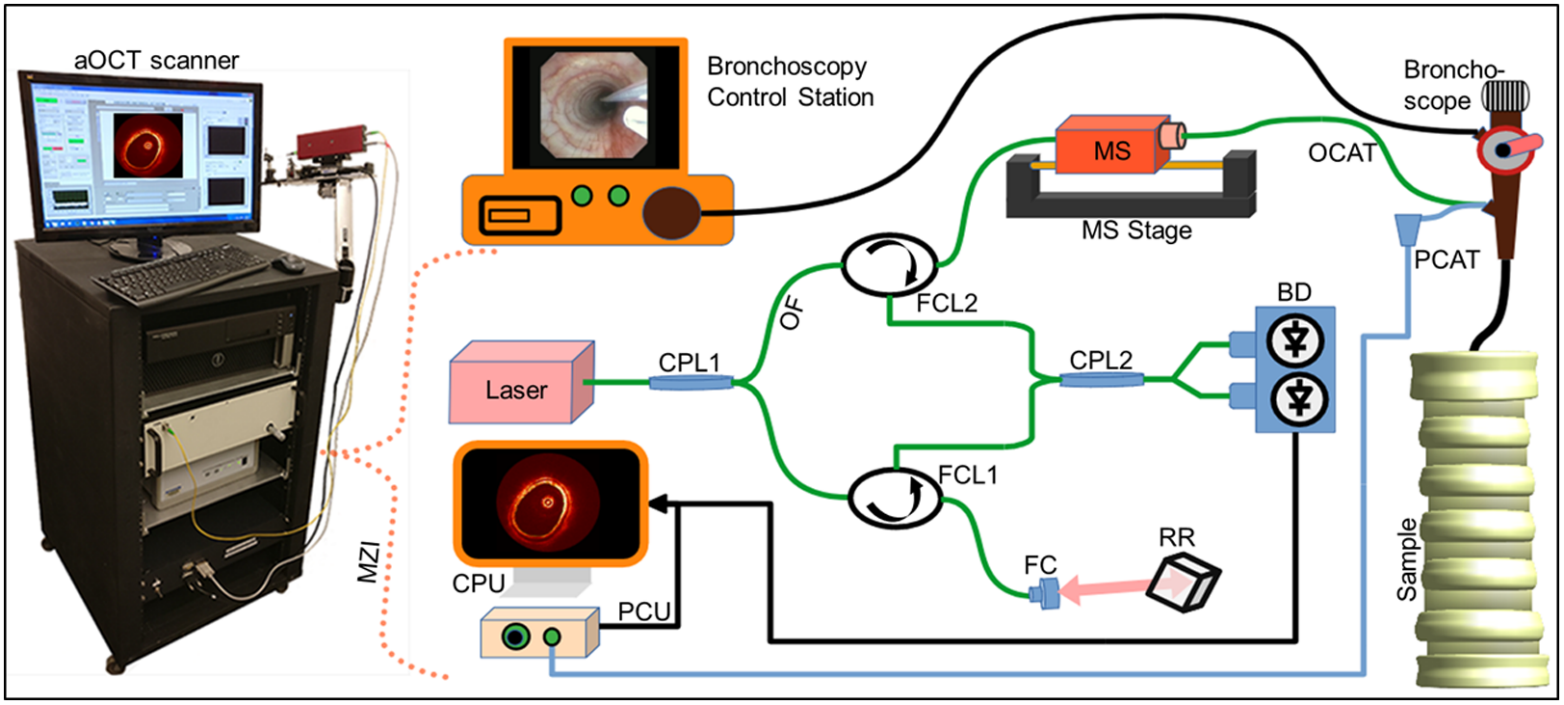
Diagram of an aOCT system with pressure catheter control unit and bronchoscopy assembly. The aOCT scanner’s Mach–Zehnder interferometer components include the optical fiber (OF), optical imaging catheter (OCAT), pressure catheter (PCAT), fiber optic circulator (FCL), fiber coupler (CPL), retro-reflector (RR), balanced detector (BD), pressure control unit (PCU), central processing unit (CPU), fiber collimator (FC), and mechanical scanner (MS).

The pressure and aOCT data acquired during 4D scanning experiments are processed using custom MATLAB scripts as follows. aOCT images corresponding to each optical imaging catheter (OCAT) rotation, shown in Fig. 2, are computed from the raw interference data using a method for digital dispersion compensation^18^ and averaging every four A-lines to reduce data overhead, for a final image size of 1249×1472 pixels (circumference × radius). Radial distances in the aOCT images were calibrated by translating a mirror at the OCAT output and accounting for the slightly off-axis (81.5 deg) angle of the beam exiting the OCAT relative to the true perpendicular. The air–tissue interface from each aOCT image was then segmented using a semi-automatic segmentation method described elsewhere.^7^ The segmentation masks were used to compute the CSA of the lumen for each image. Pressure data were averaged within the time frame of each image’s acquisition to provide co-registered data of pressure and CSA (p, CSA) for each image. The (p, CSA) data were originally collected using a sawtooth waveform in *z* programmed into LabView with the scan parameters as defined above. However, the true axial motion of the catheter was slightly different than expected. To calibrate the axial position, scans of the notched cone described below were used to compute the true *z* for each frame number. In addition, we observed an unexpected modulation in the CSA data that coincided with the turnaround points (sawtooth peaks and valleys). The cause of the modulation may be due to changes in strain in the fiber and fiber junction (compressive strain during push and tensile strain during pull) that introduce a periodic shift in the sample arm length; such a shift would induce a periodic shift in the OCT A-line and subsequently the measured CSA. To mitigate this source of noise, we performed scans of a rigid, uniform tube with constant CSA, and inferred the sample arm shift distance from apparent changes in CSA over the scan, which was typically 0.2 to 0.3 mm. Accounting for this push–pull shift in subsequent scans was successful at suppressing, but not completely eliminating, this source of modulation.

Then, (*p*, CSA) data were sorted according to the corrected *z* into bins of width Δz=0.5  mm. Ideally, the numerical simulation ensures the capture of at least one frame during inspiration (high pressure) and expiration (low pressure) within each bin. However, the obtained data did not always reflect this property, especially for bins near the beginning and end of the sawtooth scan. After rejecting frames collected at intermediate pressures between inspiration and expiration (as explained above), we excluded bins that did not contain at least 1 frame above and below the intermediate pressure range (i.e., at least one inspiratory and one expiratory pressure). After this exclusion, CC was fitted by the slope of a linear regression of the (p, CSA) data, and the uncertainty in CC was taken as the standard error of the slope for each bin. Occasionally, in regions of low CC, the regression line slope was negative; these bins were excluded.

### 2.3 System Hardware

To perform AWE, the aOCT scanner, pressure acquisition system, bronchoscope, and ventilator were carefully coordinated for consistency and repeatability. The custom aOCT system used in this study is a swept-source OCT system adapted from a system described previously.^9^ The system is comprised of a vertical-cavity surface-emitting laser (VCSEL) wavelength-swept source (SL1310V1, Thorlabs Inc., Newton, New Jersey, United States) with a wavelength sweeping range of 120 nm centered at 1310 nm, and 100 kHz repetition rate (comprising the aOCT system’s A-line rate). At the heart of the aOCT system (Fig. 2) is a fiber-optic Mach–Zehnder interferometer. The mechanical scanner (MS) is operated at a 20 Hz rotation rate such that each rotation comprises ∼5000 A-lines to form an image frame. The MS also provides pullback/push forward sawtooth scanning of the OCAT relative to the OCAT’s sheath with a total available pullback distance of ∼85  mm. The light exits the OCAT tip in a direction 81.5 deg from the forward direction (tracing out a circle as the OCAT rotates) with a power of ∼12  mW. The beam focus is ∼2 to 3 mm from the OCAT tip. The interference signal is digitized by the ATS9360, Alazar Tech. Inc. digitizer at a rate of 1.8 GS/s.

The transverse resolution of the OCT system is estimated to be 16  μm at an imaging distance of 3 mm, enlarging to 44  μm at a distance of 10 mm. The system has a measured axial resolution of 11  μm over the imaging range of ∼12  mm from the OCAT tip (corresponding to the maximum imaging radius when the OCAT is rotated). The aOCT system’s sensitivity at ∼2  mm distance is 105 dB, rolling off to 55 dB at 8.5 mm primarily due to the divergence of the beam from the focus.

The pressure acquisition system consists of a pressure control unit (PCU 2000, Millar Inc., Houston, Texas, United States), which uses a Mikro-Cath 825-0101 (Millar Inc.) catheter pressure transducer. The laser’s built-in k-clock signal is used for synchronized data acquisition of aOCT and pressure data sequences, where the trigger signal to the pressure digitizer (USB 1608FS Plus, Measurement Computing Inc., Norton, Massachusetts, United States) is reduced to 5 kHz using a custom divider circuit. Both OCAT and pressure catheter (PCAT) are introduced into the sample via a bronchoscope (Olympus EVIS EXERA III CLV 190) via a 2 mm access channel. A mechanical ventilator (900C, Siemens Servo) is used with pressure-controlled ventilation (PCV) set to a maximum inspiratory pressure (MIP) between ∼10 and 25 cm $H_{2}O$ and a ventilation rate of 20 BPM for experiments on *in vivo* pigs and inflatable samples. A custom LabView program (Physical Sciences, Inc.) was employed to simultaneously control the mechanical scanner, aOCT data acquisition, and pressure data acquisition, while also providing a GPU-accelerated real-time display of aOCT images to assist in catheter placement during experiments in conjunction with the view from the video bronchoscope.

### 2.4 3D Printed Cone for Geometric Validation

**Fig. 3:**
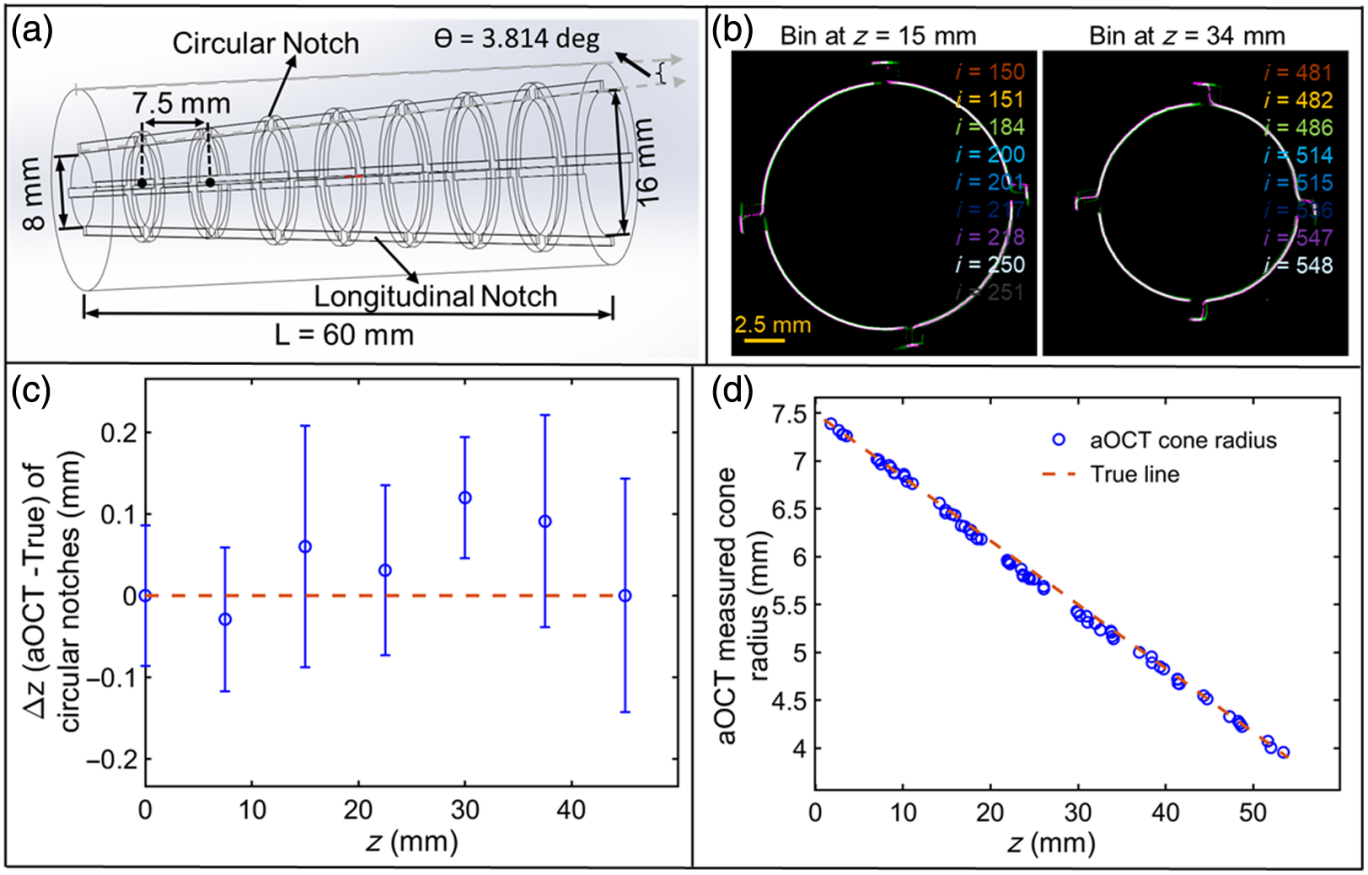
(a) Diagram of a 3D printed cone of 60 mm length with a half angle of 3.814 deg, and circular and longitudinal notches used for validating geometry obtained during 4D (sawtooth) aOCT scanning. (b) Color-coded aOCT image overlay stack of bins at z=15 and 34 mm as an example showing the grouped image frames containing matching cone radii. (c) Differences in the circular notch locations of the cone obtained by 4D aOCT compared with the true notch location. (d) Plot of cone radius (both aOCT-derived and true values) versus pullback distance. The angle of the cone retrieved from the slope of a linear fit (not shown) was $\tan^{-1}$ (0.0668) = 3.82 deg.

A rigid, hollow, conical imaging target with notches along the interior was fabricated to validate the 3D geometry obtained using the proposed 4D aOCT scanning methods. Details of the cone are shown in Fig. 3(a). The cone was 3D printed with polycarbonate-like materials (Accura 5530, Proto Labs, Inc., Maple Plain, Minnesota, United States), with a tolerance of 0.05 mm laterally and 0.127 mm longitudinally. The cone’s features provide validation of the aOCT scan z position (by its circular notches), the aOCT image radius (by the change in the cone’s radius over z), and the aOCT image orientation (by the longitudinal notches).

The OCAT was carefully positioned parallel to the central axis of the cone for aOCT imaging to ensure the observed cross sections were circular (and not elliptical). 4D aOCT scans were performed using sawtooth scan parameters $f_{\text{wfm}}=0.5\;\mathrm{Hz}$, v=6  mm/s, and α=0.6, which took a total scan time of $T_{\text{tot}}=42\;s$ along the distance $z_{\text{tot}}=50\;\mathrm{mm}$ with $N_{\text{frm}}=840$. Note that these parameter values are identical to those used in simulations in Sec. 2.1. During this rigid sample scan, there was no pressure cycling nor pressure measurement as our goal was to validate the spatial accuracy of 4D scanning after the binning process.

### 2.5 Deformable Sample for 4D Validation

Two types of deformable samples were employed to further validate the 4D aOCT method. First, a uniform hollow cylinder composed of polydimethylsiloxane (silicone) was employed to determine the precision (repeatability) of CC measurements along *z*. One end of the tube is sealed to minimize air leakage during ventilation. The tube is 93.9 mm in length with an inner diameter of 14.8 mm and a wall thickness of 1.45 mm at zero inflation pressure. For experiments, the open end of the tube is sealed to an inflated cuffed endotracheal tube (ETT), and the other side of the ETT is connected to a double swivel adapter (DSA). One of the DSA entrances is connected to the ventilator, and the other entrance is used to insert and then adjust the catheter position via the bronchoscope. The ventilator is set to operate with MIP between 8 and 35 cm H_2_O under a PCV setting with a 20 BPM respiration cycle. 4D aOCT scans were collected using the same parameters as for the rigid cone.

In addition to the silicone tube, a commercially available latex balloon that is nonuniform along its length was used to semi-quantitatively validate that heterogeneity in CC along *z* can be captured by the 4D aOCT method. The shape of the balloon is nonuniform, with narrow sections exhibiting lower compliance than wide sections. The method for mounting, ventilating, and optically scanning the balloon was identical to that for the silicone tube.

### 2.6 4D Imaging of *In Vivo* Pigs

To investigate the ability to measure the CC of live airway tissue, 4D aOCT scanning was performed on two live pigs. The experimental protocols used were approved by the Institutional Animal Care and Use Committee at the University of North Carolina, Chapel Hill. The animals were anesthetized using propofol/isoflurane and intubated before the experiments. Subsequently, vecuronium was administered to induce neuromuscular blockade, and imaging was conducted while the animals were under mechanical ventilation. Throughout the experiment, veterinary staff monitored the animals’ vital signs. The measurements were performed under PCV with the MIP set to 20 cm $H_{2}O$ and a respiratory rate of 20 BPM. The physician first examined the placement of the pig’s endotracheal tube and visually assessed the pig’s airway to position the bronchoscope. Both OCAT and PCAT were introduced through the bronchoscope’s working channel. The physician adjusted the catheter placement by looking at the live aOCT display to avoid contact with the airway. Once the catheter placement was satisfactory, a 42 s, 50 mm long sawtooth pullback scan was acquired with the same parameters as for the rigid cone. For pig 1, the scan started at the carina and ended at the mid-trachea, whereas for pig 2, the scan spanned from the primary bronchi to the mid-trachea. In the CC calculations, we excluded frames where the OCAT made contact with airway mucus or the airway wall, or exhibited obscured lumen boundaries that were difficult to resolve for an accurate CSA extraction. For pig 1, this led to the exclusion of frames 1 to 104 and 128 to 134 due to mucus contact. In addition, frames 610, 614, 615, 643, 644, 665, and 677 were excluded because of an apparent particle adhered to the catheter’s sheath, which obscured the airway lumen boundaries. Similarly, in the pig 2 scan, frames 149 to 158, 189 to 209, 218 to 224, and 243 to 259 were omitted due to the lumen area at an airway branching point extending beyond the OCT imaging range.

## 3 Results

CC is a circumferentially averaged measurement of elasticity that can reveal airway wall heterogeneity in the longitudinal direction of the specimen’s lumen. 4D scan results are shown for a static 3D imaging target, a deformable silicone thick-walled tube, and a structured balloon, to demonstrate precision in recovering lumen shape and corresponding CC. Finally, 4D imaging in pigs *in vivo* is reported to demonstrate the scanner’s ability to capture the airway’s heterogeneous compliance in a translational setting.

### 3.1 Static 3D Geometry Validation

To validate the 3D geometry reconstructed from a 4D sawtooth scan, a 3D printed, rigid, hollow cone shown in Fig. 3(a) with sets of evenly spaced axial and circular notches was used. During 4D scanning, the tip of the OCAT followed the sawtooth waveform shown in Fig. 1 such that the catheter crossed each location in *z* multiple times. Knowing the input parameters of the sawtooth waveform, we predicted the catheter’s position in time along *z* and performed binning of the collected images. Figure 3(b) shows a color-coded overlay of eight segmented images within bins at z=15 and 35 mm as a supporting example. As expected, we observed qualitatively good overlay between the segmentations derived from images collected within the same bins in *z*. We note that a small rotation in the axial notch locations between z=15 to 34 mm arises due to a noninteger number of A-lines per rotation of the OCAT; these rotations are small (∼5 to 10 deg) and do not impact the results presented here. In addition, Fig. 3(c) illustrates the difference in the circular notch positions retrieved from the sawtooth scan compared with their true positions. We find that the maximum differences are less than the 0.5 mm bin size, as expected.

As a final consistency check, we compared the cone radius extracted from segmented aOCT images (purple circles) with the true cone radius (dashed line in red) as a function of *z*, as depicted in Fig. 3(d). The standard deviation of the difference in the true and extracted cone radius is 0.11 mm. In addition, we computed the experimentally derived cone angle from the slope of a best-fit line (not depicted) and found it to be $\tan^{-1}$ (0.0668) = 3.824 deg, which is <1% different from the true cone angle of 3.814 deg. In summary, the results in Fig. 3 demonstrate that the 4D aOCT scanning method precisely retrieves 3D spatial features.

### 3.2 Precision of CC Measurements in a Uniform Tube

**Fig. 4:**
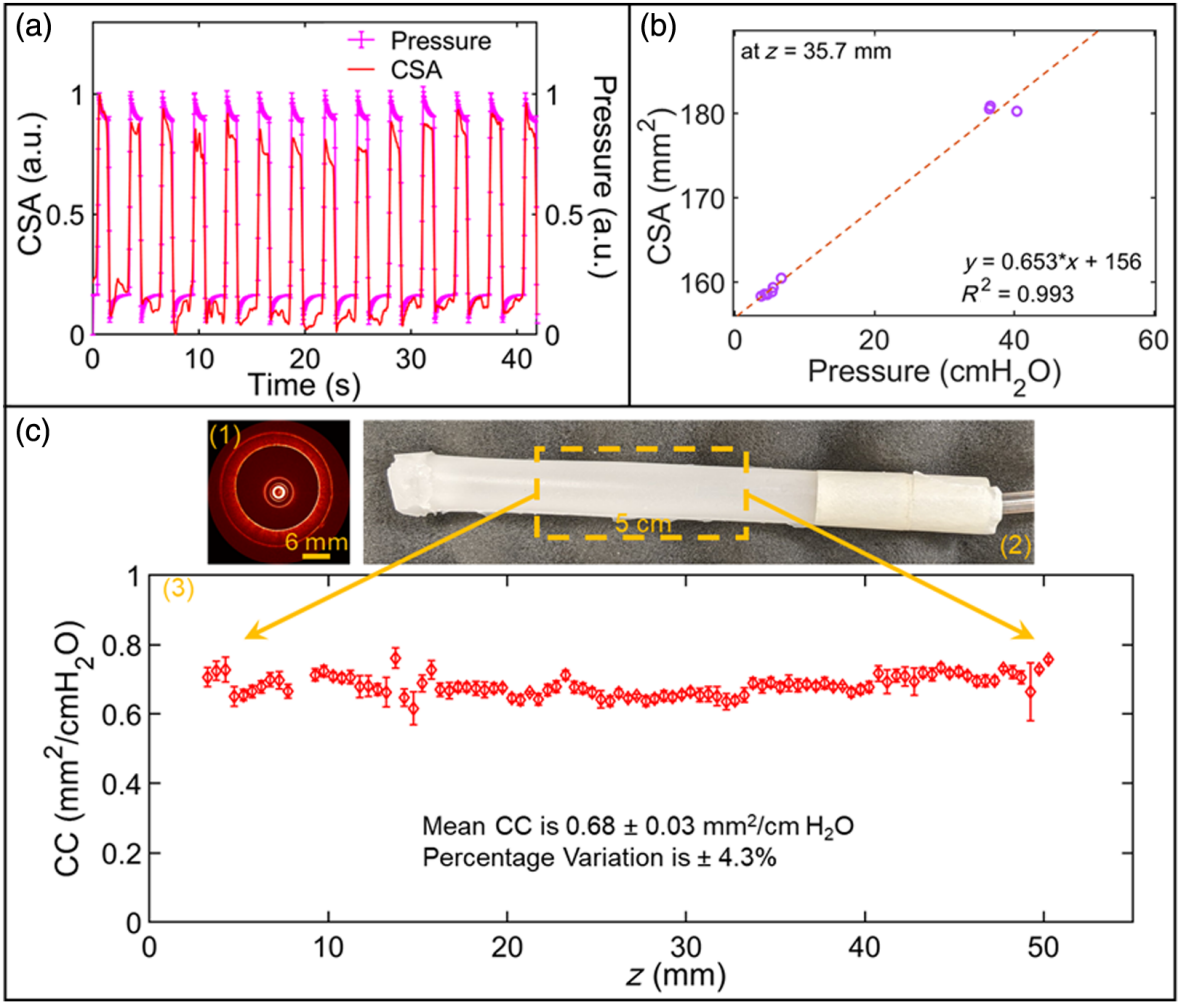
Results of 4D aOCT of a uniform silicone tube under mechanical ventilation. (a) Normalized CSA and pressure versus time demonstrate temporal synchronization. (b) Example CC calculation from linear fitting of CSA versus pressure within the bin centered at z=35.7  mm. (c) Corresponding CC along a 50 mm pullback scan of the silicone tube (bottom plot), which was collected from the dashed rectangular yellow box shown in the photograph (upper right). An example aOCT image of the tube is shown in the upper left.

To determine the precision of CC measurements using 4D aOCT, a deformable, thick-walled, silicone tube is employed with nominally uniform elastic properties. A representative aOCT image is depicted in the upper left inset of Fig. 4(c), showing both the inner and outer surface of the tube. Figure 4(a) shows (normalized) CSA and pressure data together for the duration of the scan, where the peaks and valleys are well-correlated in time.

This correlated behavior is expected for this purely elastic tube (compared with a viscoelastic material, where there would be a time delay between stress and strain), and thus, our findings suggest good temporal synchronization between intraluminal pressure and aOCT image acquisition.

Figure 4(b) shows a representative example of the linear regression used to compute CC within each bin. The values of $R^{2}$ from the regression fittings over the scan length of the tube were 0.98±0.02 (mean ± std.). Importantly, the CC values across the length of the tube, plotted in Fig. 4(c), were $0.68\pm 0.03\;{\mathrm{mm}}^{2}/\mathrm{cm}$ $H_{2}O$ (percentage variation of ±4.3%). This result indicates high repeatability of the CC measurements of the silicone tube (which itself is not perfectly uniform). However, there are some limitations evident in the plot. A lack of CC values at z∼9  mm is due to the elimination process described in the methods, where bins containing insufficiently diverse pressure data are omitted from the analysis. The region at z=13  mm exhibits a slight deviation from over all CC trend, caused by a defect (bend) in the catheter sheath impacting CSA measurement in these regions.

### 3.3 Heterogeneous Compliance of a Structured Balloon

**Fig. 5:**
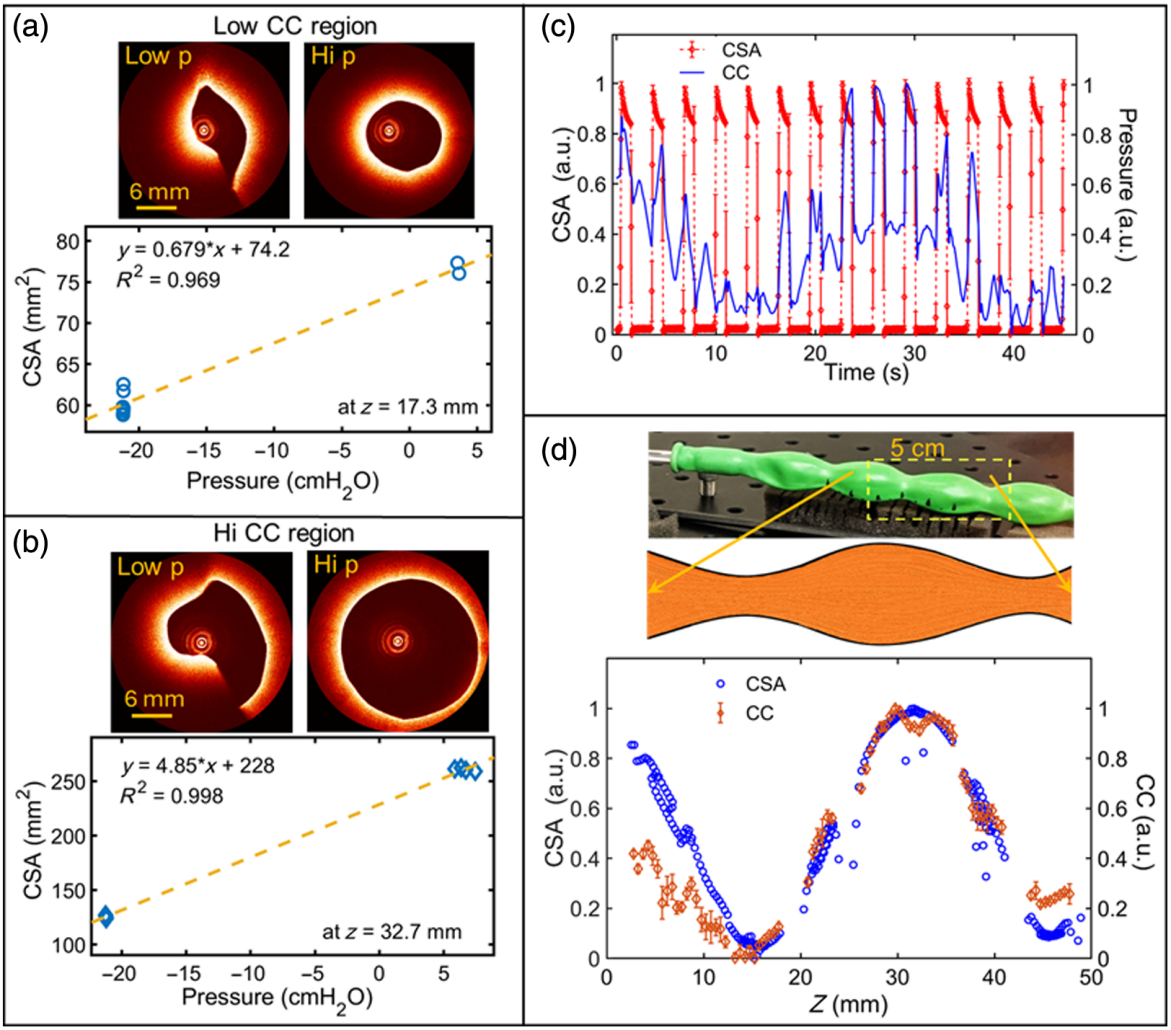
4D aOCT scan of a ventilated, structured balloon. (a) Example CC measurement for bin in a low CC region of the balloon (z=17.3  mm), with example aOCT images captured at low and high pressure. (b) Example CC measurement for bin in a high CC region of the balloon (z=32.7  mm), with example aOCT images captured at low and high pressure. (c) Normalized CSA and pressure versus time. (d) Normalized CSA and CC measurement along *z*, corresponding with the 5 cm scanned region of the object (upper diagram and photograph). CSAs plotted here are those obtained at high pressure.

To explore the capability of the proposed 4D aOCT method to capture heterogeneous CC, a commercially available, nonuniform, latex balloon is used. The balloon has a regular pattern of sections with high and low radius and correspondingly high and low compliance along its axis [Fig. 5(d)]. This means that during ventilation, the crest region of CSA shown in Fig. 5(d) is expected to exhibit high compliance, whereas the trough of low CSA, where the rubber is pinched, is expected to exhibit low compliance.

Example CC calculations are displayed in Figs. 5(a)–5(b) for low compliance and high compliance regions. The difference in CC between these regions is clearly visible as a difference in the slope of the CSA versus pressure regression lines. The raw pressure and CSA data versus time [Fig. 5(c)] depict a strong correlation between peaks in the applied stress (pressure) and resultant strain (CSA); however, unlike the uniform tube, the peak strain values vary across the scan due to the balloon’s nonuniformity. The average value of $R^{2}$ from the regression fitting over the entire length of the balloon was ±0.25 (mean ± std.), indicating reasonable linearity between the measured stress and strain. Most importantly, the CC varies as expected across the length of the balloon, with high CC in regions of high CSA, and low CC in regions of low CSA [Fig. 5(d)]. As in the uniform tube, gaps in the CC data near z=20, 25, 35, and 42 mm correspond to bins lacking a high-pressure image (with corresponding gaps in the high CSA data in the plot). We also note that in some of the low-pressure images, such as those depicted in Figs. 5(a) and 5(b), the CSA may have been underestimated due to the folding of the balloon’s surface obstructing the laser’s line-of-sight, creating hidden regions. Overall, this effect does not appear to influence the expected shape of the CC trace.

### 3.4 Compliance of *In Vivo* Pig Upper Airways

**Fig. 6:**
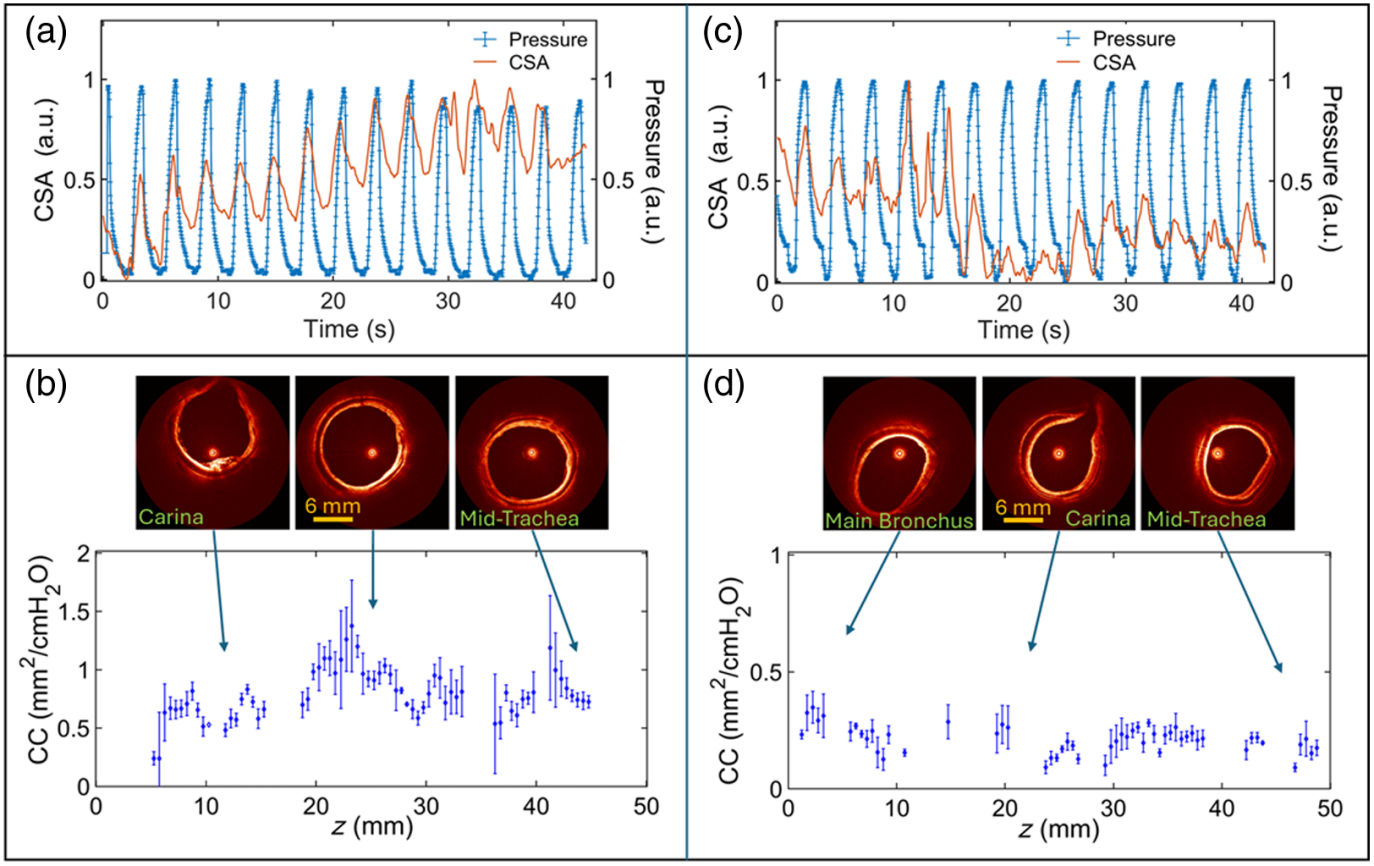
4D aOCT scans from two different live pigs. (a) Normalized CSA and pressure versus time for pig 1. (b) Corresponding CC measurement from carina to mid-trachea of pig 1. The inset aOCT images are from the beginning, middle, and end of the scan (Video 1). (c) Normalized CSA and pressure versus time for pig 2. (d) Corresponding CC measurement from the main bronchus or primary bronchi to the carina of pig 2. The inset aOCT images are from the initial, middle, and end of the scan (Video 2), (Video 1, mp4, 6.7 MB [URL: https://doi.org/10.1117/1.JBO.30.12.124502.s1]; Video 2, mp4, 6.7 MB [URL: https://doi.org/10.1117/1.JBO.30.12.124502.s2] at 4×).

To investigate the ability to measure CC of the airway wall during a clinic-like bronchoscopy exam, 4D aOCT scanning was performed on two live, anesthetized pigs under mechanical ventilation. The results shown in Figs. 6(a) and 6(c) display a high degree of synchronization between peaks of CSA and corresponding peaks in pressure over the duration of both scans. Figures 6(b) and 6(d) show the resulting CC of the pig airway walls over the lengths of their 50 mm scans; the scan for pig 1 (left) starts at the carina and pulls back to the mid-trachea (see video 1 at 4× real time), whereas the scan for pig 2 (right) starts at the primary bronchi and pulls back to the mid-trachea (see video 2 at 4× real time). For pig 1, there is a lack of CC data below z=5  mm and at z=11, 27, and 34 mm due to the omission of frames where the OCAT contacted the airway wall and/or mucus. Similarly, for the pig 2 scan in Fig. 6(d), the gaps in CC data are due to the omission of frames where airway lumen segmentation was not possible, including frames with an airway branch that extended beyond the 12 mm imaging radius of the OCT system.

For pig 1, CC ranged from 0.24 to $1.37\;{\mathrm{mm}}^{2}/\mathrm{cm}$ $H_{2}O$, and for pig 2, CC ranged from 0.05 to $0.35\;{\mathrm{mm}}^{2}/\mathrm{cm}$ $H_{2}O$. These *in vivo* values are reasonably consistent with previously reported CC measurements using an ordinary pullback aOCT scan on *ex vivo* excised pig tracheas, which ranged from 0.63 to $1.52\;{\mathrm{mm}}^{2}/\mathrm{cm}$ $H_{2}O$.^10^ Compared with the CC measurements from the silicone tube and the uniform balloon, the live pig airways exhibited a larger variation, with a value of 27% (mean and std. in $R^{2}$ is 0.79±0.22) for pig 1 and 28% (mean and std. in $R^{2}$ is 0.74±0.26) for pig 2. This is expected as the pig airways may generally be heterogeneous and give rise to natural variations along the pullback.

## 4 Discussion

To the best of our knowledge, this is the first demonstration of a retrospective, respiratory-gated (3 + 1)D anatomical OCT scanning method. The method as applied here is shown to capture airway deformation at various phases of the respiratory cycle across a 50 mm length of the airway, which, when combined with intraluminal pressure measurement, is used to quantify the CC along the length of the airway with a sampling resolution of 0.5 mm (for a total of 100 CC measurements). Importantly, 4D scans as proposed only require 42 s to acquire in a clinically relevant scenario, which is favorable toward translation to clinical bronchoscopy, effectively requiring 0.42 s per CC measurement. In comparison, a standard pullback scan can, at best, provide a single measurement of CC over a respiratory cycle (≥3  s per CC measurement), which is prohibitive for assessing a significant portion of the airway during bronchoscopy. The proposed method thus constitutes a significant advance in our ability to perform AWE via minimally invasive aOCT.

In this first demonstration, the sawtooth scan parameters are developed in simulation using the anticipated respiratory rate of the subject to ensure that samples are collected at both high and low pressures within each bin. These scan parameters are then implemented in the software controlling the scanner. A possible weakness of this approach is if the subject is breathing spontaneously or is being ventilated at a different rate. This could be mitigated in future approaches by dynamically adjusting the sawtooth scan for the respiratory rate in real time, the latter of which could be readily determined from the intraluminal pressure data. In addition, the current protocol does not compensate for possible longitudinal motion of the airway relative to the imaging probe that is associated with cyclical airway stretching (and elongation) during respiration, which is a limitation that may affect the spatial consistency of CC measurements. It may be possible to account for such repetitive stretching effects in future work with appropriate modeling.

Another possibility with this method is to quantify the viscoelasticity of the airway wall, as in prior aOCT work,^9^ by tracking hysteresis in the pressure-CSA curves. However, this would likely require longer scans to ensure sampling a greater diversity of pressures to accurately estimate the hysteresis loops.

The 4D scanning method demonstrated here is already capable of quantifying CC over a significant portion of the airway that will be of interest in several areas of biomedicine. One interest area is for assessment of upper airway obstructive disorders, where abnormal airway compliance is implicated in surgical failures and existing methods are lacking. Another immediate application is for assessing and monitoring airway injury due to smoke inhalation (burn), and axially resolved CC over the length of the airway may be useful to assess the extent and severity of injury.

## 5 Conclusion

The aOCT-based retrospective, respiratory-gated 4D scanning method provided measurements of airway wall cross-sectional compliance (CC) over a 50 mm scan length in both a deformable tube model and *in vivo* pigs. Initially, calibration of the scanning process was achieved using a 3D-printed cone, and specific features such as cone angle aligned well with the true cone dimensions. To quantify the method’s precision, we scanned a compliant uniform rubber tube and obtained a variation of ±4.2% in CC. Then, a structured balloon was scanned to demonstrate the ability to detect elastic heterogeneity, where the computed CC along the length of the balloon clearly mapped regions of low and high compliance [Fig. 5(d)]. Finally, 4D aOCT scans were conducted on two *in vivo* pigs [Figs. 6(b)–6(d)], to assess the approach in a simulated clinical senario. The results showed CC values similar to previous reports and displayed distinct variations in CC between the pigs, highlighting the potential of this minimally invasive, retrospective, respiratory-gated aOCT method for AWE.

## 6 Appendix: Video Captions

The following videos are mentioned in the text:

Video 1 *In Vivo* 50 mm scan at 4X (mp4, 6.7 MB [URL: https://doi.org/10.1117/1.JBO.30.12.124502.s1]).

Video 2 *In Vivo* 50 mm scan at 4X (mp4, 6.7 MB [URL: https://doi.org/10.1117/1.JBO.30.12.124502.s2]).

## Supplementary Material

10.1117/1.JBO.30.12.124502.s1

10.1117/1.JBO.30.12.124502.s2

## Data Availability

Code and data can be made available upon request to the corresponding author.

## References

[1] Wong B. J. F. et al., “In vivo optical coherence tomography of the human larynx: Normative and benign pathology in 82 patients,” Laryngoscope 115(11), 1904–1911 (2005). 10.1097/01.MLG.0000181465.17744.BE 16319597

[2] Ajose-Popoola O. et al., “Diagnosis of subglottic stenosis in a rabbit model using long-range optical coherence tomography,” Laryngoscope 127(1), 64–69 (2017). 10.1002/lary.26241 27559721 PMC5326617

[3] Armstrong J. J. et al., “Quantitative upper airway imaging with anatomic optical coherence tomography,” Am. J. Respir. Crit. Care Med. 173(2), 226–233 (2006). AJCMED 1073-449X 10.1164/rccm.200507-1148OC 16239620

[r4] Jing J. et al., “High-speed upper-airway imaging using full-range optical coherence tomography,” J. Biomed. Opt. 17(11), 110507 (2012). JBOPFO 1083-3668 10.1117/1.JBO.17.11.110507 23214170 PMC3494494

[r5] Wijesundara K. et al., “Quantitative upper airway endoscopy with swept-source anatomical optical coherence tomography,” Biomed. Opt. Express 5(3), 788 (2014). BOEICL 2156-7085 10.1364/BOE.5.000788 24688814 PMC3959831

[r6] Jing J. C. et al., “Anatomically correct visualization of the human upper airway using a high-speed long range optical coherence tomography system with an integrated positioning sensor,” Sci. Rep. 6, 39443 (2016). SRCEC3 2045-2322 10.1038/srep39443 27991580 PMC5171831

[7] Price H. B. et al., “Geometric validation of continuous, finely sampled 3-D reconstructions from aOCT and CT in upper airway models,” IEEE Trans. Med. Imaging 38(4), 1005–1015 (2019). ITMID4 0278-0062 10.1109/TMI.2018.2876625 30334787 PMC6476567

[8] Walsh J. H. et al., “Evaluation of pharyngeal shape and size using anatomical optical coherence tomography in individuals with and without obstructive sleep apnoea,” J. Sleep Res. 17(2), 230–238 (2008). JSRSEU 1365-2869 10.1111/j.1365-2869.2008.00647.x 18422508

[9] Balakrishnan S. et al., “Combined anatomical optical coherence tomography and intraluminal pressure reveal viscoelasticity of the in vivo airway,” J. Biomed. Opt. 23(10), 100501 (2018). JBOPFO 1083-3668 10.1117/1.JBO.23.10.100501 30350490 PMC6259006

[10] Bu R. et al., “Airway compliance measured by anatomic optical coherence tomography,” Biomed. Opt. Express 8(4), 2195 (2017). BOEICL 2156-7085 10.1364/BOE.8.002195 28736665 PMC5516819

[11] Bu R. et al., “Localized compliance measurement of the airway wall using anatomic optical coherence elastography,” Opt. Express 27(12), 16751 (2019). OPEXFF 1094-4087 10.1364/OE.27.016751 31252896 PMC6825607

[12] Bu R. et al., “Sensing inhalation injury-associated changes in airway wall compliance by anatomic optical coherence elastography,” IEEE Trans. Biomed. Eng. 68(8), 2360–2367 (2021). IEBEAX 0018-9294 10.1109/TBME.2020.3037288 33175676 PMC8110609

[13] Williamson J. P. et al., “Elastic properties of the central airways in obstructive lung diseases measured using anatomical optical coherence tomography,” Am. J. Respir. Crit. Care Med. 183(5), 612–619 (2011). AJCMED 1073-449X 10.1164/rccm.201002-0178OC 20851930

[14] McLaughlin R. A. et al., “Respiratory gating of anatomical optical coherence tomography imaging of the human airway,” Opt. Express 17(8), 6568–6577 (2009). OPEXFF 1094-4087 10.1364/OE.17.006568 19365482

[15] Goss B. C. et al., “Magnetic resonance elastography of the lung: technical feasibility,” Magn. Reson. Med. 56(5), 1060–1066 (2006). MRMEEN 0740-3194 10.1002/mrm.21053 17036283

[16] Jiang J. H. Turner J. F. Huang J. A., “Endobronchial ultrasound elastography: a new method in endobronchial ultrasound-guided transbronchial needle aspiration,” J. Thorac. Dis. 7, S272–S278 (2015). 10.3978/j.issn.2072-1439.2015.12.53 26807274 PMC4700375

[17] Blocker S. J. et al., “The impact of respiratory gating on improving volume measurement of murine lung tumors in micro-CT imaging,” PLoS One 15(2), e0225019 (2020). POLNCL 1932-6203 10.1371/journal.pone.0225019 32097413 PMC7041814

[18] Marks D. L. et al., “Digital algorithm for dispersion correction in optical coherence tomography for homogeneous and stratified media” Appl. Opt. 42(2), 204–217 (2003). APOPAI 0003-6935 10.1364/AO.42.000204 12546500

